# Sinopharm (HB02)-associated vaccine-induced immune thrombotic thrombocytopenia: a case report

**DOI:** 10.1186/s13256-023-04086-7

**Published:** 2023-09-08

**Authors:** Mohammad Sistanizad, Tahereh Sabaghian, Hossein Amini, Fahimeh Hadavand, Mahmood Nabavi, Mehran Kouchek, Mir Mohammad Miri, Sara Salarian, Seyedpouzhia Shojaei, Omid Moradi

**Affiliations:** 1 Department of Clinical Pharmacy, School of Pharmacy, Shahid Beheshti University of Medical Sciences, Tehran, Iran; 2 Prevention of Cardiovascular Disease Research Center, Shahid Beheshti University of Medical Sciences, Tehran, Iran; 3 Division of Nephrology, Department of Internal Medicine, Shahid Beheshti University of Medical Sciences, Tehran, Iran; 4 Infectious Diseases and Tropical Medicine Research Center, Imam Hossein Teaching and Medical Center, Shahid Beheshti University of Medical Sciences, Tehran, Iran; 5 Department of Infectious Diseases, Imam Hossein Teaching and Educational Center, Shahid Beheshti University of Medical Sciences, Tehran, Iran; 6 Department of Pulmonary and Critical Care Medicine, Imam Hossein Teaching and Educational Center, Shahid Beheshti University of Medical Sciences, Tehran, Iran; 7 Department of Clinical Pharmacy, Faculty of Pharmacy, Hormozgan University of Medical Sciences, Bandar Abbas, 7919691982 Iran

**Keywords:** COVID-19, Vaccine, Thrombosis, Whole-virus vaccine, Case report

## Abstract

**Background:**

Vaccine-induced thrombotic thrombocytopenia is associated with the coronavirus disease 2019 vaccines. It has been reported by vector-based vaccines. To the best of our knowledge, there is no report about vaccine-induced thrombotic thrombocytopenia in whole-virus vaccines. We are presenting the first case of vaccine-induced thrombotic thrombocytopenia with this type of vaccine.

**Case presentation:**

An 18-year-old male Caucasian patient with complaints of severe abdominal, low back, and lower extremity pain presented to the medical center. He received the first dose of the Sinopharm (HB02) vaccine against coronavirus disease 2019 10 days before hospital attendance. In the laboratory examination, decreased platelet count and increased D-dimer were observed. During hospital admission, the diagnosis of pulmonary embolism was reached. He received vaccine-induced thrombotic thrombocytopenia therapy consisting of intravenous immune globulin and direct oral anticoagulant. Platelet count increased and he was discharged after 1 month.

**Conclusion:**

This case highlights the possibility of vaccine-induced thrombotic thrombocytopenia occurrence by whole-virus coronavirus disease 2019 vaccines. Compared with vector-based vaccines, this phenomenon is rare for whole-virus vaccines. More studies on this type of vaccine regarding thrombotic thrombocytopenia should be considered.

## Background

The coronavirus disease 2019 (COVID-19) pandemic has been affecting the global population since December 2019. Vaccines are being used worldwide to combat the pandemic and reduce deaths associated with the disease [1]. Different types of vaccines, including Messenger ribonucleic acid (mRNA), vector-based, whole-virus, and protein subunit, are being administered [2].

Sinopharm, an inactivated whole-virus vaccine developed by Chinese scientists, produces immunity against severe acute respiratory syndrome coronavirus 2 (SARS-CoV-2) severe infection [3]. Previous studies on the vaccine report tolerable adverse drug reactions, including injection site reaction, fever, etc., without reporting severe adverse reactions [4].

A thrombotic event associated with vaccines against COVID-19 was first reported in February 2021, alongside thrombocytopenia in individuals who received ChAdOx1 nCov-19. Afterward, this serious adverse reaction was classified as vaccine-induced immune thrombotic thrombocytopenia (VITT) [5, 6]. Reported cases received adenovirus vector-based COVID-19 vaccines. The incidence of this syndrome is extremely rare [5].

In the following case report, we describe the first case of VITT caused by an inactivated whole-virus vaccine from Iran. In this case, we report the clinical presentation, diagnosis, associated complication, and treatment approach of a VITT associated with the Sinopharm vaccine.

## Case presentation

An 18-year-old Caucasian male patient with no history of any medical condition presented to the emergency department of Imam Hossein tertiary medical center from Shahid Beheshti University of medical sciences in November 2021, complaining of fatigue, malaise, headache, severe persistent abdominal pain, and pain in the low back and lower extremities of 7-day duration before hospital admission. Reduced power in lower limbs was observed in the legs (4/5). He also reported the presence of nausea and vomiting. Symptoms associated with the genitourinary system were absent. Level of consciousness was intact. No signs and symptoms associated with cardiovascular and respiratory systems were reported. He had no history of bleeding from any sources, bruising, or thrombotic conditions. In addition, there was no history of hematologic disorders regarding bone marrow suppression. The history was negative for medicine use, except receiving the first dose of the Sinopharm (HB02) vaccine against COVID-19 10 days before presenting at the medical center. Before being admitted to the hospital, he had visited the outpatient clinic, but there was no change in his pain after receiving fluid therapy and analgesics.

In the emergency department, he was alert and oriented to person, place, and time. Hemodynamics were stable. Vital signs were in the normal range. In the examination of the head and neck, sclera was not icteric, pupils were reactive to light, mucous membranes were not dry, jugular vein was not prominent, and thyroid was of normal size and consistency. The trachea of the patient was in the middle line, the movement of the chest was similar, the lungs were clear, S1 and S2 sounds were heard during cardiac auscultation, and pathological sounds were not heard. No petechiae, rashes, or ecchymoses were observed. In the abdominal examination, he complained of abdominal tenderness. No organomegaly was observed in the physical examination of the abdomen. The neurologic findings included pain in the lumbar position and lower limbs. The force of the two lower extremities was decreased, and the deep tendon reflexes were reduced. No specific findings regarding the cardiovascular system were reported.

Laboratory reports were follows: platelet count, 104,000 cells per µL; white blood cells (WBC), 4000 cells per µL; hemoglobin (Hgb), 13.0 g/dL. The biochemistry results showed an increase in the liver transaminases (aspartate aminotransferase, 173 unit/L; alanine aminotransferase, 59 unit/L). In addition, a significant increase in creatine phosphokinase (5642 international unit/L) and lactate dehydrogenase (754 IU/L) was reported. Moreover, an increase in the inflammatory biomarkers consisting of C-reactive protein (56 mg/L) and erythrocyte sedimentation rate (22 mm/hour) was reported. Coagulation parameters were increased (prothrombin time, 17.7 seconds; international normalization ratio, 1.64). Fibrin degradation products were 20.7 μg/mL. The fibrinogen level was 339 mg/dL. D-dimer significantly increased to 2622 ng/mL. The NT-pro-BNP was enormously increased (14,391 pg/mL). Other laboratory findings regarding the serum creatinine, bilirubin, and serum electrolyte were in the normal range. The patient was evaluated for infectious disease causes. Urine analysis was normal, and cultures for urine and blood (two sets of samples) were negative. The SARS-CoV-2 polymerase chain reaction was negative. The Wright and 2ME tests were negative as well.

In the paraclinical examination, transthoracic echocardiography was performed, and left ventricular ejection fraction (LVEF) was reported as 55% and global hypokinesia with mild tricuspid regurgitation with pulmonary artery pressure of 28 mmHg was reported. No vegetation was detected in the echocardiography.

During his admission course, on the second day of hospitalization, oxygen saturation declined suddenly, and oxygen supplementation was initiated. Computed tomography (CT) angiography was performed. There was no obvious vascular filling defect, however, there was a non-enhancing wedge shape consolidation with reversed halo sign at the poster basal segment of right lower lobe (RLL) as a new finding in comparison with a previous exam that could be due to infarct at subsegmental branches. Clinical and D-dimer level correlation was recommended. In addition, he complained of severe pain and tenderness in the right arm. Doppler sonography was performed, but no sign of thrombosis was reported.

After 48 hours of hospitalization, due to the severity of symptoms, he was transferred to the intensive care unit (ICU).

In the course of the illness, the platelet count was reduced to 57,000 cells/µL. No platelet transfusion was performed. D-dimer reached 2820 ng/mL. A slight increase in coagulation tests was observed. Unfortunately, a laboratory test for confirming heparin-induced thrombocytopenia (heparin-induced platelet activation) was unavailable in the medical center.

**Fig. 1:**
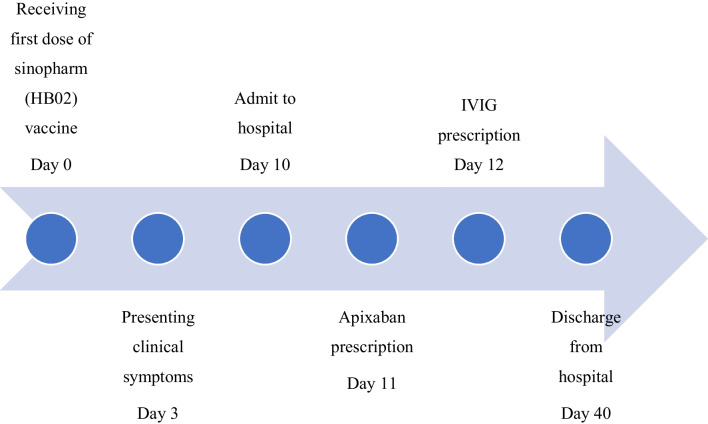
Patient timeline

Due to the appearance of symptoms after the injection of the vaccine, and having no risk factors for pulmonary thromboembolism and platelet drop, the diagnosis of VITT was possible. Patients received pharmacotherapy of VITT on the basis of the latest recommendation from the American Society of Hematologists and the International Society on Thrombosis and Haemostasis [7, 8]. In the management of the patient, anticoagulation with apixaban, a direct oral anticoagulant, was initiated. According to the pulmonary CT angiography report and high D-dimer, the treatment team decided to treat pulmonary thromboembolism, so apixaban was prescribed at a dose of 10 mg twice a day for 1 week, then 5 mg twice a day. He received intravenous immunoglobulin with a total dose of 2 g/kg given in five doses. After intravenous immunoglobulin initiation, platelet count increased and patient’s condition improved. During the ICU admission, no episode of bleeding was reported. At the time of discharge, platelet count was 293,000 cells/μL. Liver enzymes decreased but were slightly above the normal upper range. The coagulation test was normal. D-dimer was still increased (3560 ng/mL). Platelet and D-dimer timelines are presented in Table 1. At the time of discharge, the patient was advised to take apixaban in the form of 5 mg twice a day for 6 months and to return to the outpatient clinic 1 month after discharge, but the patient’s follow-up was not completed as the patient did not return. The patient’s timeline is shown in Fig. 1.

**Table 1:** Platelet and D-dimer timelines

Timeline day (first day of admission, day 10)	Day 10	Day 14	Day 16	Day 40 (discharge)
Platelet (× 10^3^/μL)	104	57	110	293
D-dimer (ng/mL)	2622	2830	–	3560

## Discussion and conclusion

The exact pathophysiology of the VITT is still unknown. Different types of COVID-19 vaccines were associated with this condition, including adenovector-based and mRNA vaccines [5, 9]. Here we report a case of a whole-virus vaccine. Virus components, including proteins and genome that bind to the platelet factor 4 (PF4), may induce autoantibody formation, platelet activation, and thrombosis formation. Production of high titer of antibody against PF4 after vaccination followed up by platelet aggregation and granulocyte activation is the main pathophysiology behind VITT [10].

Presentation and timing of the initiation of symptoms in our patient were compatible with the other cases of VITT previously reported [11]. Systemic symptoms alongside thrombocytopenia, pulmonary thrombosis, and increased FDP and D-dimer levels were all compatible with VITT diagnosis. To confirm the diagnosis, positive result and detection of autoantibodies against platelet factor 4 with an ELISA kit in a symptomatic patient are recommended, and the result alone is not sufficient [8].

In this particular case, the most important finding was the type of vaccine. To date, no cases of VITT were reported due to the Sinopharm vaccine as a whole-virus-based COVID-19 vaccine. In addition, we observed the thrombosis formation in pulmonary vasculature but not cerebral vein thrombosis, which is the most common position of thrombosis formation in the previously reported cases [11].

There is a chance of VITT associated with the whole-virus vaccine, but further confirmatory investigation is needed to assess the exact risk of this condition. To evaluate the pathogenesis and involved mechanism of this serious adverse effect and determine the responsible part of the vaccine, more cases must be evaluated, and a complete laboratory test performed.

## Data Availability

The supporting for this report is available on request from the corresponding author.

## Abbreviations

COVID-19: Coronavirus disease 2019
SARS-CoV-2: Severe acute respiratory syndrome coronavirus 2
VITT: Vaccine-induced immune thrombotic thrombocytopenia
WBC: White blood cells
LVEF: Left ventricular ejection fraction
CT: Computed tomography
ICU: Intensive care unit
IVIG: Intravenous immune globulin
